# A novel approach for combining the metagenome, metaresistome, metareplicome and causal inference to determine the microbes and their antibiotic resistance gene repertoire that contribute to dysbiosis

**DOI:** 10.1099/mgen.0.000899

**Published:** 2022-12-20

**Authors:** Vitalii Stebliankin, Musfiqur Sazal, Camilo Valdes, Kalai Mathee, Giri Narasimhan

**Affiliations:** ^1^​ Bioinformatics Research Group (BioRG), Knight Foundation School of Computing and Information Sciences, Florida International University, Miami, FL, USA; ^2^​ Herbert Wertheim College of Medicine, Florida International University, Miami, FL, USA; ^3^​ Biomolecular Sciences Institute, Florida International University, Miami, FL, USA; ^†^​ Present address: Microsoft Corporation, GA, Atlanta, USA; ^‡^​ Present address: Lawrence Livermore National Laboratory, 7000 East Avenue, Livermore, CA 94550, USA

**Keywords:** antibacterial resistance, causal Bayesian network, multi-omics, origin of replication, peak-to-trough ratio (PTR)

## Abstract

The use of whole metagenomic data to infer the relative abundance of all its microbes is well established. The same data can be used to determine the replication rate of all eubacterial taxa with circular chromosomes. Despite their availability, the replication rate profiles (metareplicome) have not been fully exploited in microbiome analyses. Another relatively new approach is the application of causal inferencing to analyse microbiome data that goes beyond correlational studies. A novel scalable pipeline called MeRRCI (Metagenome, metaResistome, and metaReplicome for Causal Inferencing) was developed. MeRRCI combines efficient computation of the metagenome (bacterial relative abundance), metaresistome (antimicrobial gene abundance) and metareplicome (replication rates), and integrates environmental variables (metadata) for causality analysis using Bayesian networks. MeRRCI was applied to an infant gut microbiome data set to investigate the microbial community’s response to antibiotics. Our analysis suggests that the current treatment stratagem contributes to preterm infant gut dysbiosis, allowing a proliferation of pathobionts. The study highlights the specific antibacterial resistance genes that may contribute to exponential cell division in the presence of antibiotics for various pathogens, namely *Klebsiella pneumoniae, Citrobacter freundii, Staphylococcus epidermidis, Veilonella parvula* and *Clostridium perfringens *. These organisms often contribute to the harmful long-term sequelae seen in these young infants.

## Data Summary

The code developed for this manuscript is available via a Github link from our website: http://biorg.cs.fiu.edu/merrci/.

The authors confirm that all supporting data, code and protocols have been provided within the article or through supplementary data files.

**Impact Statement**

This paper presents a novel pipeline for microbiome analysis. Prior efforts have focused on computing eubacterial community and antibiotic resistance genes (metaresistome) profiles from whole metagenome sequencing (WMS) data. This study expands the field by introducing the estimation of replication rates of the eubacteria with circular chromosomes (metareplicome) of the microbiome. In addition, it demonstrates how causal discovery techniques can be applied to the combination of omics profiles and clinical data to study survival strategies against antibiotics. The proposed pipeline was used to re-analyse a preterm infant gut microbiome dataset. The preterm infants were administered a variety of antibiotics during their early months. The analyses highlighted the role of antibacterial resistance (ABR) genes in bacterial survival strategies used by different eubacteria in the microbiome to respond to the administered antibiotics. The study also highlighted several infant and maternal variables that affected eubacterial dysbiosis. The study suggests that discontinuing the use of some antibiotics (such as ticarcillin-clavulanate and ceftriaxone for infants and mothers, respectively) and targeting the genes that appear to allow a particular pathogen to proliferate may help prevent many of the acute and adverse long-term sequelae seen among preterm neonates.

## Introduction

Globally, antibiotic resistance (ABR) has emerged as one of the biggest threats to public health [1]. In the USA alone, over 2.8 million people get severe infections from antibiotic-resistant pathogens, resulting in about 35 000 deaths annually [2]. Many new antibiotics were designed to target different bacterial pathways. Nevertheless, their efficacy wanes over time as microbes develop resistance to counter the drugs [3]. In addition, exposure to antibiotics irreversibly changes microbiota composition and host response to some microbial signals [4]. Such antibiotic-associated transformations have been linked to changes in the levels of hormones, colonic short-chain fatty acids and cholesterol, thus leading to increased adiposity [5]. Understanding how microbial communities respond to antibiotics is of utmost importance.

Antibiotics such as *β*-lactams target cell wall synthesis, making the dividing cells most vulnerable. Bacteria are known to evade the effect of *β*-lactam exposure by losing the cell walls [6], halting their division temporarily [7] and then resuming their normal growth at the end of the treatment [8]. The bacteria respond to antimicrobials by controlling cell division, thus, going into survival mode [9]. Some slow-growing bacteria are known to have better survival rates upon antibiotic exposure [10]. Alternatively, bacteria may respond to *β*-lactam exposure by producing *β*-lactamases (chromosomally encoded or acquired) that hydrolyse the antibiotics providing resistance [11]. The expression of *β*-lactamases can increase with increasing growth rates, allowing bacteria to continue to multiply even in the presence of some antibiotics [12]. Antibiotics could also increase the replication rates of harmful bacteria, which can be potentially detrimental to the host [13]. Thus, the replication rate could serve as an indicator to track ABR and better understand eubacterial defence mechanisms against antibiotics. Interestingly very little is known about the replication rates in microbiomes and how exposure to antibiotics influences the process.

Traditionally, whole metagenome sequencing (WMS) data have primarily been used to compute the relative abundance of each microbe in a microbiome [14, 15]. However, knowing the replication rates of each microbe in the sample can shed light on microbiota dynamics. Recent work has made it possible to use the same whole metagenomic data to calculate the replication rates of each eubacterium with circular chromosomes in a microbiome sample [16]. Replication rates are inferred from WMS by exploiting the mechanism of bacterial bidirectional replication of circular chromosomes, which starts at the origin of replication (*oriC*) and elongates in both directions of the DNA strand until it reaches the terminus (*dif* site) [17, 18]. Thus, if bacteria are actively growing, more reads will be observed near the origin than the termination of replication. The average number of replication forks per cell for a given bacterial genome can be measured by determining what is called the peak-to-trough ratio (PTR), which is the ratio of the coverage near *oriC* to the coverage near *dif* [16, 19]. This method is available as an open-source software called bPTR, which includes automatic detection of the origin of replication based on GC-skew [20]. The bPTR analysis demonstrated that bacterial replication rates of some species were correlated with ulcerative colitis [16], necrotizing enterocolitis [20] and Crohn’s disease [21]. The bacterial replication rates depend on the sampling site [21, 22] and bacterial taxonomy [20] but are independent of proteome composition [23]. In addition, replication rates can provide insights into microbe responses to antibiotics. Bacterial taxa can lower their replication rates in response to antibiotics [23, 24] and resume their growth after cessation of the exposure [20, 24, 25]. The average bacterial replication rate was correlated with the quantity of ABR genes found in a sample [25].

Even though some studies simultaneously measured the replication rates of several microorganisms [26, 27] from metagenomic samples, interactions within a microbial community were not considered in the analysis. Unfortunately, such omissions ignore synergies between species that play a vital role in microbiome functioning and can reveal new properties not observed when studied in isolation [28]. For example, it has been shown that resident microbial communities can suppress the growth and evolution of pathogenic *Escherichia coli* [29]. In addition, *Streptococcus* species recruit other microbes to colonize various niches of the oral cavity [30].

Currently, microbiome analytical tools primarily focus on obtaining associational or correlational information [31]. However, associations do not necessarily imply causation, leaving a critical gap in understanding complex biological processes and mechanisms as a collection of causal relationships between the variables in the microbiome. The human microbiota-associated (HMA) model is a well-known laboratory-based approach for establishing causal relationships between microbiome and host phenotype, where faecal microbiota from human cohorts are transplanted to germ-free mice [32, 33]. While HMA models work well to identify disease-associated microbial species, discovering critical microbial interactions or other mechanisms causally linked to the phenotype remains a challenge [34]. Host-associated genetic and other environmental variables can be confounding factors in HMA models. Multi-omics studies, such as the integrated Human Microbiome Project (iHMP), allow for a comprehensive view of microbiome and host factors that influence dysbiosis [35]. Causal links within the microbiome can be predicted from such observational studies using the Bayesian Networks approach [36]. Despite challenges related to confounding factors and limited sample sizes, Bayesian Network analysis has been shown to successfully eliminate accidental correlations and select the strongest associations within the microbiome [36, 37]. Although computational causality prediction tools are available, they have not been applied to microbiome data sets, extensively.

The approach proposed here allows us to study the growth behaviour of all eubacteria with circular chromosomes of a broader microbial community under antibiotic exposure, which is better than exploring the response of isolated eubacterial taxa [10, 12, 38, 39]. In a diverse microbiome, different microbes employ different strategies to deal with the antibiotics in the environment that determine the microbiome composition post-treatment. This study presents a novel approach of using computational causal inferencing on a multi-omics microbiome data set that includes the metagenome (eubacterial relative abundance), metareplicome (eubacterial replication rates) and metaresistome (profile of antibacterial resistance genes), along with metadata (clinical variables). Metagenomic sequence data from a longitudinal study of sampled preterm infants [40] were re-analysed with bacterial replication rates of each taxon in the microbiome to discern the impact of antibiotics and resistance development.

## Methods

### MeRRCI, a scalable pipeline

**Fig. 1.**
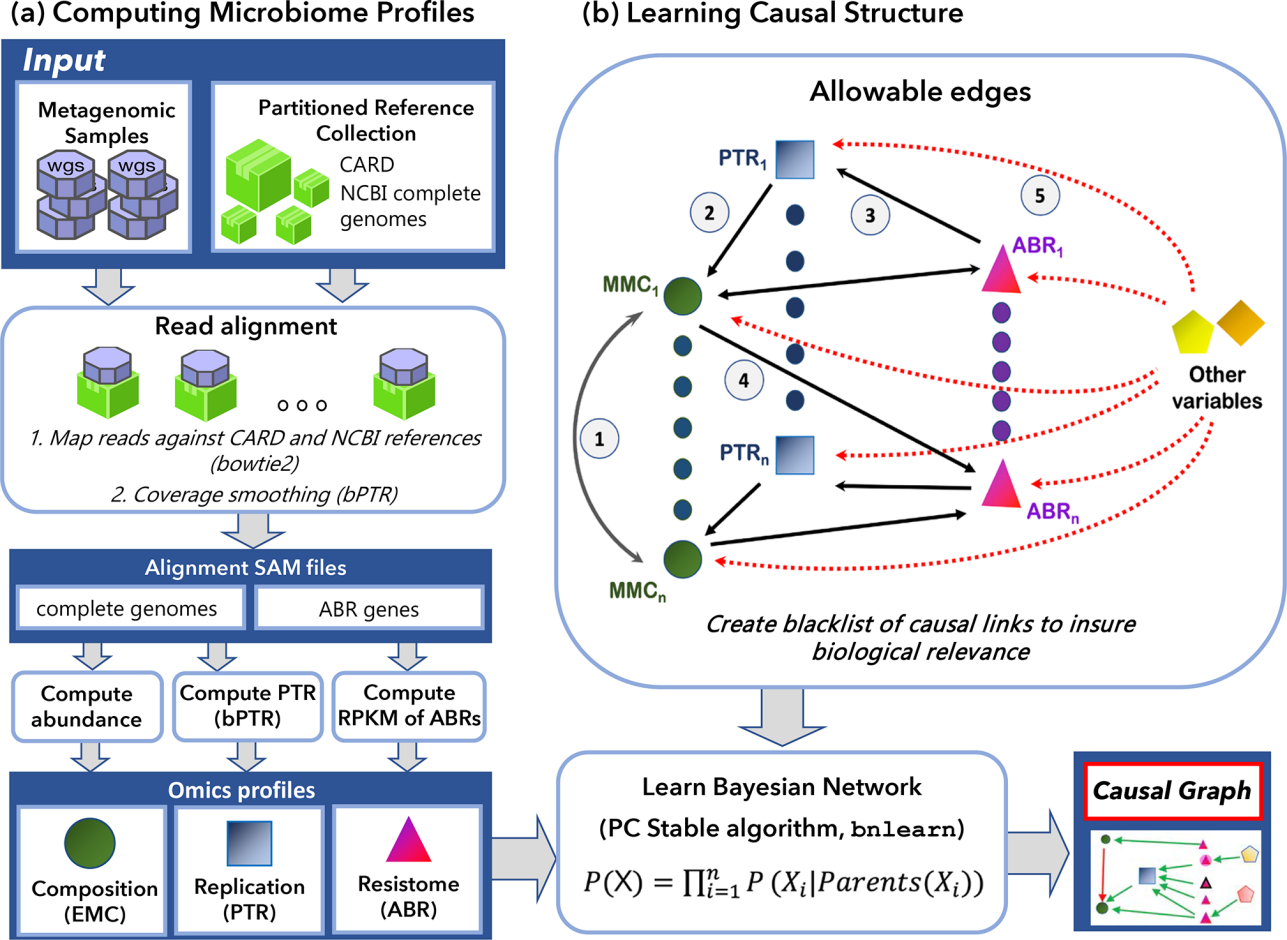
MeRRCI – an approach to infer consequences of antibiotic resistance from whole metagenomic studies. (a) A high-performance computing framework for computing microbiome-wide eubacterial metagenomic composition (EMC), eubacterial replication rates as the peak to trough ratio (PTR), and profiles of antibiotic resistance (ABR) genes. The reference collection of genome sequences is partitioned into smaller subcollections. Mapping reads to the database of genome sequences and resistance genes are achieved by using bowtie2, resulting in alignment files in SAM format. The setup is well suited for parallelization and the use of high-performance computing environments. The output of this phase includes the three omics profiles – EMC, PTR and ABR. (b) The tool bnlearn can infer causal relationships between the variables of interest. The inputs to this tool are the omics profiles from the previous phase and a set of constraints with the list of allowable edges. These constraints incorporate our biological understanding of the microbiome and its interactions with the host. The output of this phase is a causal graph where the directed edges are causal relationships that were inferred computationally. The constraints are pictured as a network model containing edges that are allowed to be present. Note that different shapes are used for different types of nodes. The allowed types of edges are shown numbered 1–5. Edges of type 1 connect nodes representing taxon relative abundance; edges of type 2 go from replication rates (PTR) of taxa to their relative abundance; edges of type 3 lead from gene expression to replication rate nodes; type 4 nodes connect gene abundance and taxa; type 5 edges connect miscellaneous variables to all other types of nodes. All other edges are disallowed and included in a blacklist.

In this study, a novel, two-phase, scalable pipeline called Metagenome, metaResistome and metaReplicome for Causal Inferencing (MeRRCI) was developed (Fig. 1). Phase A involves computing three profiles from the raw sequence data, namely eubacterial metagenomics composition (EMC), ABR gene composition, and eubacterial replication rate as the PTR (Fig. 1a). A causal structure discovery algorithm was applied in the second phase to discern relationships between the variables from the three computed profiles (EMC, ABR and PTR) and measured clinical variables (Fig. 1b). The pipeline was executed on a high-performance multiprocessor computing environment with the Slurm resource management system [41].

#### MeRRCI phase A: computing EMC, ABR and PTR

A previously developed pipeline, FLINT, for metagenomic profiling was adapted with minor modifications [42]. In FLINT, the Map-Reduce procedure breaks down the set of sequencing read into smaller subsets. Reference genome sequences are distributed for smaller collections allowing for high-performance parallel computations. The resulting pipeline allows scalability for processing a vast number of metagenomic reads and the use of extensive reference genome collections. Mapping is achieved using bowtie2, an ‘ultrafast and memory-efficient tool for aligning sequencing reads to long reference sequences’, resulting in sequence alignment maps (SAMs) [43]. In this study, we enhanced FLINT to include (a) mapping of resistance genes against a comprehensive antibiotic resistance database (CARD) [44] and (b) computing eubacterial replication rates as described below. The output of this phase is the EMC, PTR and ABR quantitative profiles.

The publicly available comprehensive, non-redundant and well-annotated NCBI Reference Sequence database RefSeq v.92 was used to compute the EMC and PTR profiles. Only complete (or closed) genomes were selected to ensure the quality of the reference database used, resulting in 30 382 microbial genomes. For optimal use of FLINT, those reference genomes were split into 64 roughly equal-sized partitions to create bowtie2 genome indexes [42, 45]. The indexes were loaded onto the 64 different computational nodes on the high-performance computing cluster. Thus, each read was sent to all the 64 nodes, which run with their local index containing a subset of genomes from the repository. Our previous work on FLINT showed that partitioning the index into any number of clusters yields the same abundance profiles as with a single large reference [42]. Since each read is mapped against every partition and the counts are accumulated, the number of partitions does not change the computed counts in MeRRCI.

Each node used bowtie2 to align every read to its local index partition with a default minimum score threshold [43]. Mixed and discordant alignments were prohibited to ensure the pair-end constraints (*--no-discordant* and *--no-mixed* options). Each alignment was counted independently when a read aligned to several reference sequences. The smoothing sliding window approach from bPTR software was applied to address uneven coverage probably caused by multi-mapping to shared-sequence regions [20]. In particular, if the coverage value for a 100 bp window differed more than eight-fold from *m,* the median value over 1000 neighbouring windows, the coverage was replaced by *m*. The alignment for a particular reference was discarded as a ‘false positive’ if more than 2 % among all sliding windows had a coverage correction. However, even with such smoothing filtering, some false positives can be missed due to multi-mapping reads, a common challenge in metagenomics [46]. The output alignment SAM files were used to compute the EMC, PTR and ABR profiles.

The normalized average coverage was used as the measure for EMC. The average coverage, *C*, of a given genome was computed by the following formula:

$$C=\frac{NL}{G}\;\;\;\;\;\;\;\;\;\;\;(1)$$

where *N* is the total number of reads that align to the given genome sequence, *L* is the average length of a metagenomic read aligning to that genome and *G* is the length of that genome sequence. The relative abundance of each genomic taxon was aggregated on a species level and normalized to add up to 1.

The average number of replication forks per cell for a given bacterial genome was determined by computing the PTR, the ratio of the coverage near *oriC* to the coverage near *dif* [16, 19]. Those genomes with an average coverage of fewer than five reads per base (5× coverage) were filtered out to obtain reliable PTR values [20]. To the best of our knowledge, no studies have provided an estimate of the PTR error as a function of genome coverage. Based on a previous study, genomes with average coverage larger than 5× are sufficient for accurate estimation of replication rates [16]. The alignment files for filtered reference genomes were used to compute PTR with bPTR software [20]. For this study, species-level PTR values were obtained using the following formula:

$$PTR_{s}=\frac{\sum_{i=1}^{N}PTR_{si}\alpha_{si}}{\sum_{i=1}^{N}\alpha_{si}},\;\;\;\;\;\;\;\;\;\;(2)$$

where *PTR_s_* is the PTR value for species *S* with *N* strains; *PTR_si_* is the PTR value of strain *i* of species *S*; and *α*_*si*_ is the relative abundance of strain *i* of species *S*. Such an aggregation gives the average PTR value for a given species *S* and reduces their variance providing more robust statistical results. The weighting eliminates anomalies that arise from extremely high PTR values of strains with low relative abundance.

The metaresistome profiles were computed by mapping the metagenomic reads against CARD version 3.0.7, which has 2624 ABR genes [47]. As a proxy for the antibiotic resistance ‘potential’ of the metagenomic sample, reads per kilobase per million mapped reads (RPKM) of reference resistance genes were calculated from the alignment files. Genes were considered present if metagenomic reads covered at least 80 % of its nucleotides along with their reference, which is the most used threshold for positive ABR gene identification [48, 49]. The same EMC alignment settings were used to generate ABR profiles.

#### Phase B: causality analysis

In the second phase, potential causal relationships within a microbiome were inferred by modelling the data as a Bayesian Network using a state-of-the-art causal discovery algorithm called PC Stable included in the bnlearn R package [50–52]. The input to this tool is the EMC, PTR, ABR and metadata.

The causal structures (or causal Bayesian networks) are defined as a class of probabilistic graphical models where each vertex (node) represents one of *n* random variables from a set, *X* = {*Xi,i=1, …, n*}, and each edge represents an inferred (direct) causal relationship [53, 54]. These structures are represented as a directed graph *G* = (*V, E*), where each vertex (or node) in *V* represents a random variable from *X*, and *E* is the set of edges (or links) that connect the nodes. Undirected edges are used in cases where the direction cannot be reliably determined or both directions appear to be valid. In most causal inferencing algorithms, the graph *G* is often ‘manipulated’ to be a Directed Acyclic Graph (DAG) [55]. Each random variable *X_i_* has an associated probability distribution, denoted by *P* (*X_i_*).

A directed edge in *E* between two vertices represents direct stochastic dependencies. Therefore, if there is no edge connecting two vertices, the corresponding variables are either marginally or conditionally independent (conditional on some subset of the rest of the variables). The ‘local’ probability distribution of a variable *X_i_* depends only on itself and its parents (i.e. the vertices with directed edges into the node *X_i_*); the ‘global’ probability distribution *P* (X) is the product of all local probabilities, i.e. a joint distribution [50], as in

$$P\left(X\right)=\prod_{i=1}^{n}P\left(X_{i}|Parents\left(X_{i}\right)\right)(3)$$

This equation is straightforward when the causal structure is sparser. Thus, an essential step in our pipeline is to identify all independent pairs of random variables that represent indirect or non-causal relationships.

All local structures in a causal network can be classified into three sub-categories: *chains*, *forks* and *colliders* [56]. In a *chain*, two variables *X* and *Y* are conditionally independent given *Z*, if the set *Z* intersects every path from *X* to *Y*. In a *fork*, a variable *Z* is a ‘common cause’ for variables *X* and *Y*; this happens when there is no directed path between *X* and *Y*, and become independent when conditioned on *Z*. Finally, a set *Z* is a ‘collider’ node between *X* and *Y*, if it is the ‘common-effect’. In a *collider*, as in the fork, there is no directed path between *X* and *Y*. However, the difference is that *X* and *Y* are unconditionally independent but become dependent when conditioned on *Z* or the descendants of *Z*. A directed graph *G* has two vertices, *χ* and *y,* that are *d*-connected if and only if *G* has a collider-free path connecting *χ* and *y* [57]. Meaningful relationships inferred from causal Bayesian networks constructed using principles of *d*-separation can be more informative than association analysis. A directed edge from *X* to *Y* in such networks suggests that changing *X* will cause a change in the value of *Y* even if all other variables are held constant [58]. Therefore, with probabilistic graphical models, it is possible to infer one variable’s causal impact, assuming that there are no hidden confounders. Even if the real data include hidden confounders, the model’s approximations give valuable insights into causal relationships in a multivariate data set.

The set of allowable edges was restricted as described below to ensure biologically relevant causal structures (Fig. 1b). Such restrictions increase the robustness of the Causal Bayesian network and help to exclude coincidental correlations [59, 60]. The restrictions are as follows:

##### Type 1

Taxon abundance → Taxon abundance. The Type 1 edges represent how much the relative abundance of one eubacterium impacts the abundance of another, thus possibly representing interactions, such as cooperation or competition [61].

##### Type 2

PTR → abundance (of the same taxon). The Type 2 edges represent the extent to which a change in the replication rate of a taxon may alter its relative abundance.

##### Type 3

ABR gene abundance → PTR. The Type 3 edges represent the impact of the presence of a resistance gene on the replication rate of a taxon. This information allows us to infer if the presence of a particular ABR gene promotes the replication of the taxon that carries it.

##### Type 4

Taxon abundance ↔ ABR gene abundance. A Type 4 edge from a taxon relative abundance to a resistance gene may suggest that the taxon carries the genes, while the edge from a resistance gene to taxon relative abundance may indicate the importance of the given resistance gene in survival.

##### Type 5

Environmental variables → Taxon abundance, PTR, ABR gene abundance}. In the Type 5 edges, the measured clinical and environmental parameters are considered to be external variables that impact all microbiome entities but are not causally affected by them.

The network was evaluated on robustness by randomly bootstrapping samples with 100 replicates [62]. An edge between two variables was contained if its empirical frequency over the bootstrapped networks was at least 0.7. To keep the resulting models sparse and straightforward, we constructed a model using only the most abundant major bacterial species and ABR genes present in at least 5 % of the study samples.

### Cohort analysis and clinical variables

The MeRRCI pipeline was applied to a premature infant gut microbiome data set (BioProject ID: PRJNA301903) [40]. The cohort consisted of 84 premature infants, and metagenome data were generated from 401 stool samples. All but two infants received a course of antibiotics within the first 24 h. Forty-nine infants received additional antibiotic treatments between 1 and 10 weeks of life, forming the ‘Antibiotic Cohort’ (AC). The remaining 35 formed the ‘Control Cohort’ (CC). Each treatment consisted of one or more antibiotics from the following list – ampicillin (AMP), meropenem (MEM), ticarcillin-clavulanate (TIM), gentamicin (GEN) and vancomycin (VAN). Stool specimens were collected at multiple time points, with roughly 2–6 samples per infant between 6 to 156 days of life. The clinical variables measured for the infants include their age in days, birthweight, furosemide Lasix and CRIB II score measures, antibiotic dosage administered, type of milk consumed (formula, maternal or donor milk) and other dietary information (vitamin A, caffeine and iron). The clinical variables measured for the mother were the antibiotics [ampicillin (AMP), ceftriaxone (CRO), azithromycin (AZM), amoxicillin (AMX), cefazolin (CEZ), erythromycin (ERY), gentamicin (GEN), penicillin (PEN), vancomycin (VAN), clindamycin (CLI), cefotaxime (CTX)] and duration of membrane rupture of the mother prior to child delivery.

### Statistical discriminant analysis

While this work focuses on the causal discovery, the analysis was supplemented by comparing different omics measures before and after antibiotics. For example, a relative abundance and replication rates of specific bacterial taxa, quantity of specific ABR genes, and changes after specific antibiotics exposure were compared. The non-parametric Mann–Whitney U test was used for these comparisons, which allowed us to compute statistical significance [63]. The Benjamini–Hochberg *P*-value correction was applied to correct for multiple hypothesis testing, where appropriate [64].

The analysis was also supplemented by investigating the association between different omics measures. For example, the associations between bacterial replication rates and relative abundance were evaluated using the non-parametric Spearman correlation [65], which measures the strength of the monotonic relationship between paired data. The choice of the correlation method was motivated by the non-linear nature of the measured variables. The relative abundance of a taxon is expected to be an exponential function of its replication rate. The correlations were obtained using the stats standard R library [66].

Finally, the discriminatory ability of different measured variables and their significance were investigated. The partial least squares discriminant analysis (PLS-DA) [67] technique for feature selection using the MixOmics R package [68] was applied to identify the ABR genes that help to discriminate between the different antibiotics. The PLS-DA is a supervised machine learning tool that finds the direction (i.e. principal component) that maximizes class separation. Ten-fold cross-validation and ten repeats with *tune.splsda* was used to select features that contribute most to the class separation.

### Hardware

The MeRRCI pipeline was executed using the High-Performance Computing (HPC) environment at FIU’s Instructional and Research Computing Center [69]. The job submission was handled by Slurm Workload Manager version 19.05.3–2. The cluster contains 1500 Intel-based cores with high memory nodes ranging from 32 to 384 GB per node. All jobs were submitted to computational nodes with CentOS v.7 operating system. Eight threads per process were used to execute several jobs within the same computational node. Other computational steps were performed on a server-class machine at FIU (castalia) with 792 GB of RAM and 48 Intel Xeon processors.

## Results

### Eubacterial metagenomic composition (EMC)

**Fig. 2.**
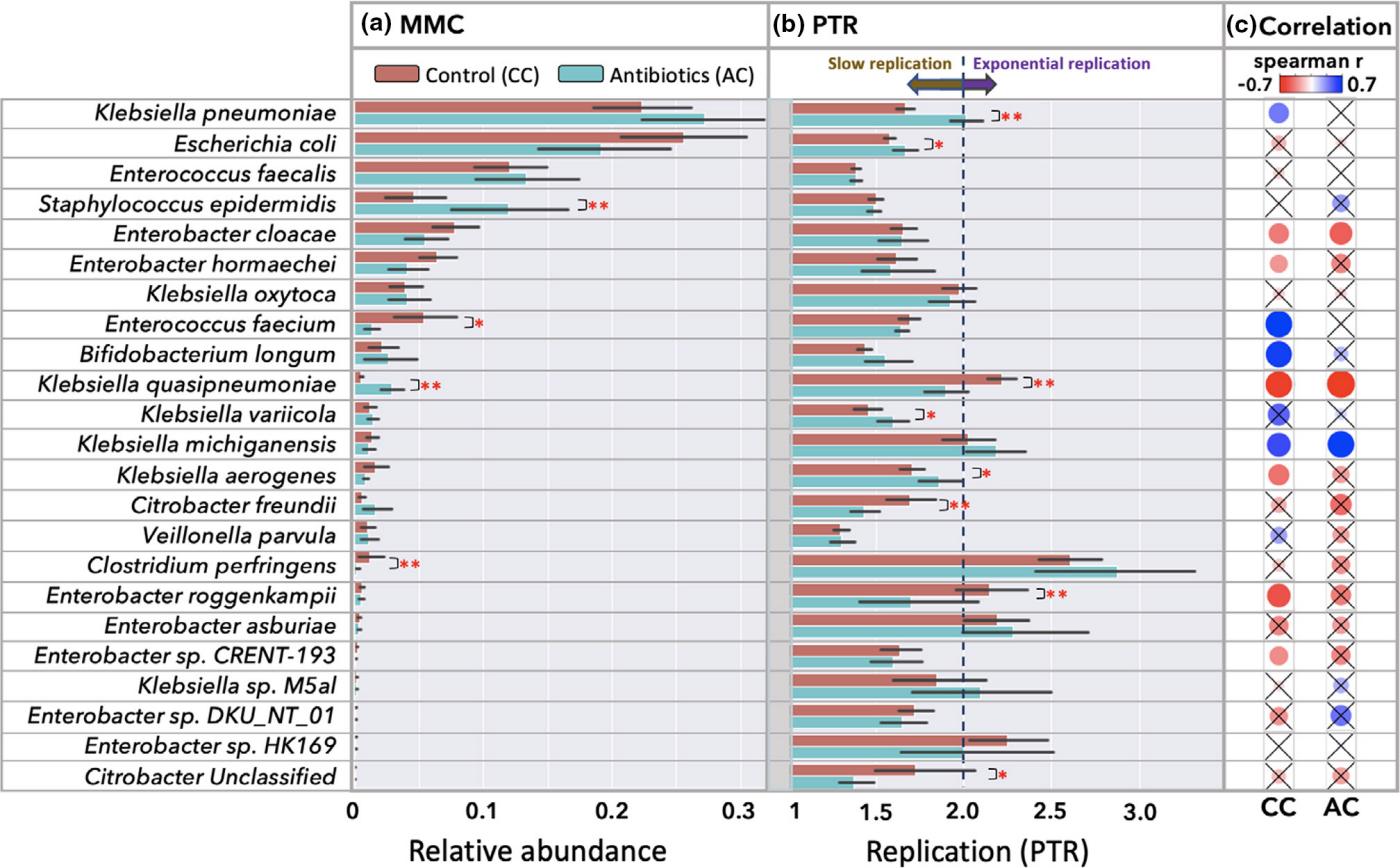
Microbial metagenomic composition (EMC) and replication rate (PTR) profiles. (**a**) Average re-normalized relative abundance for the most frequently present species found in control (CC) and antibiotic (AC) cohorts. (**b**) Average replication rates (PTR) values for bacterial species. Statistical significance was evaluated with a Mann–Whitney U test, where: *P≤0.05 and **P≤0.01. Black lines on each bar indicate standard deviation. (**c**) Spearman correlation coefficients between PTR and relative abundance values of the same taxon computed separately for the two cohorts, CC and AC. The colour of the filled circles signifies the sign of the correlation (blue for positive and red for negative). In contrast, the size of the filled circles represents the absolute value of the correlation coefficient. Crossed entries represent correlations that are not significant (*P*>0.05).

The EMC was computed as normalized average coverage of metagenomic reads mapped against reference genomes. In total, 154 bacterial species were identified. To reduce the data sparsity for robust causality predictions, only genomes with average read coverage of at least 5× and present in at least 5 % of the total samples were considered. Such filtering resulted in 25 unique eubacterial taxa (Fig. 2a). The most abundant species in both cohorts were *Klebsiella pneumoniae, Escherichia coli* and *Enterococcus faecalis. Staphylococcus epidermidis* and *Klebsiella quasipneumoniae* were significantly overabundant in the antibiotic cohort (AC), while *Enterococcus faecium* and *Clostridium perfringens* were more abundant in the control cohort (CC).

### Replication rates using peak to trough ratios (PTR)

The MeRRCI pipeline computes the replication rate by determining the ratio of the number of reads at the *oriC* with *dif* for each eubacterial taxon from metagenomic read coverage [16, 19] (Fig. 2b). PTR values above 2.0 indicate exponential replication, whereas lower values suggest the opposite [16]. *Clostridium perfringens* was the most highly replicating species in both cohorts despite their low relative abundance. In addition, *Enterobacter asburiae, Enterobacter* sp. HK169 and *K. michiganensis* were actively replicating in both cohorts. In the AC, *K. pneumoniae, K. aerogenes, Escherichia coli* and *K. variicola* had higher replication rates. In contrast, *K. quasipneumoniae, Enterobacter roggenkampii* and *Citrobacter freundii* showed significantly lower replication rates in the AC.

The Spearman correlation coefficient was calculated to determine the association’s strength and direction of the association between corresponding replication rates and relative abundance values (Fig. 2c). A strong negative correlation between replication rate and relative abundance is observed in the CC for *Enterobacter* (*Enterobacter cloacae, Enterobacter hormaechei, Enterobacter roggenkampii* and *Enterobacter* sp. CRENT-193, Column 1) and *Klebsiella* (* K. quasipneumoniae *, and *K. aerogenes *) species. However, there is a strong positive correlation between replication rates and relative abundance values for *K. pneumoniae, Enterococcus faecium, Bifidobacterium longum* and *K. michiganensis *. In the AC, only three species showed significance: negatively for *K. quasipneumoniae* and *Enterobacter cloacae* and positively for *K. michiganensis *.

### Resistome profiles

**Fig. 3.**
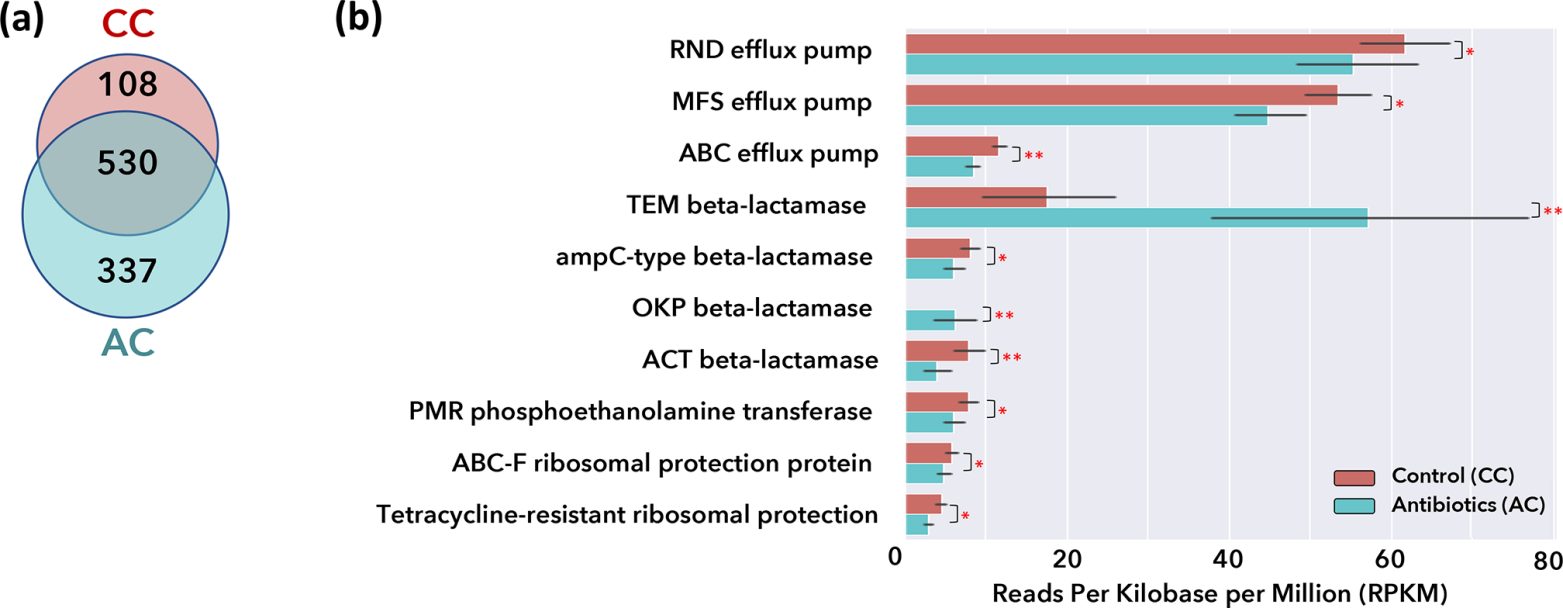
ABR resistance profile. (**a**) Venn diagram of the number of genes associated with each of the two cohorts (CC and AC) with *P*≤0.05. (**b**) Reads per kilobase million (RPKM) for the most abundant ABR gene families found in the dataset, where *P≤0.05 and **P≤0.01. Black lines on each bar indicate standard deviation. Statistical significance for (**a-b**) were established with Mann–Whitney U tests.

The MeRRCI pipeline identified ABR genes present in the dataset by mapping metagenomic reads against the CARD database [44]. A total of 975 unique ABR genes were identified, from which 108 were significantly overabundant in the CC, while 337 were associated with the AC (Fig. 3a). The top 10 most differentially abundant ABR gene families are listed in Fig. 3b. Of these, efflux pumps (RND, MFS and ABC), ampC-type and ACT *β*-lactamase, PMR phosphoethanolamine transferase, and ribosomal protection protein (ABC-F and tetracycline-resistant) were overabundant in the CC. On the other hand, TEM and OKP *β*-lactamase had significantly higher relative abundance in the AC.

### Immediate response of replication on antibiotic exposure

**Fig. 4.**
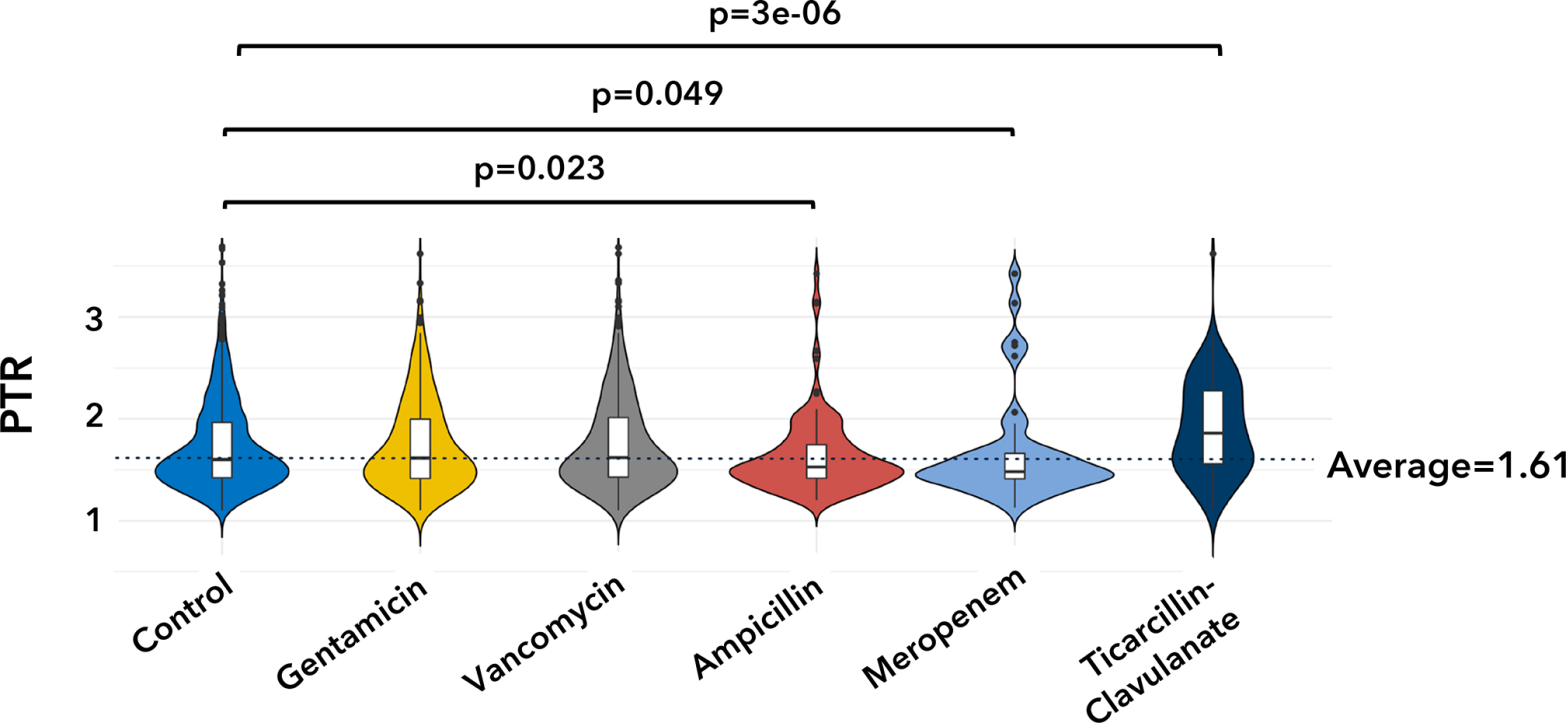
Distribution of PTR values. Violin plots showing the distribution of PTR values for taxa in the samples from the control cohort (CC) infants and infants who recently received antibiotic treatment (AC). Mean PTR in each treatment group was compared to CC samples using the Mann–Whitney U test (*P*-values are as shown in the figure).

The distribution of PTR values for each recently administered antibiotic class was compared between the CC and AC (Fig. 4). There was no significant difference between the mean PTR for the CC compared to those who were recently administered GEN or VAN. However, the average PTR values were significantly lower in the AC than the CC after the administration of MEM and AMP. The average PTR value was higher upon TIM treatment in the AC.

**Fig. 5.**
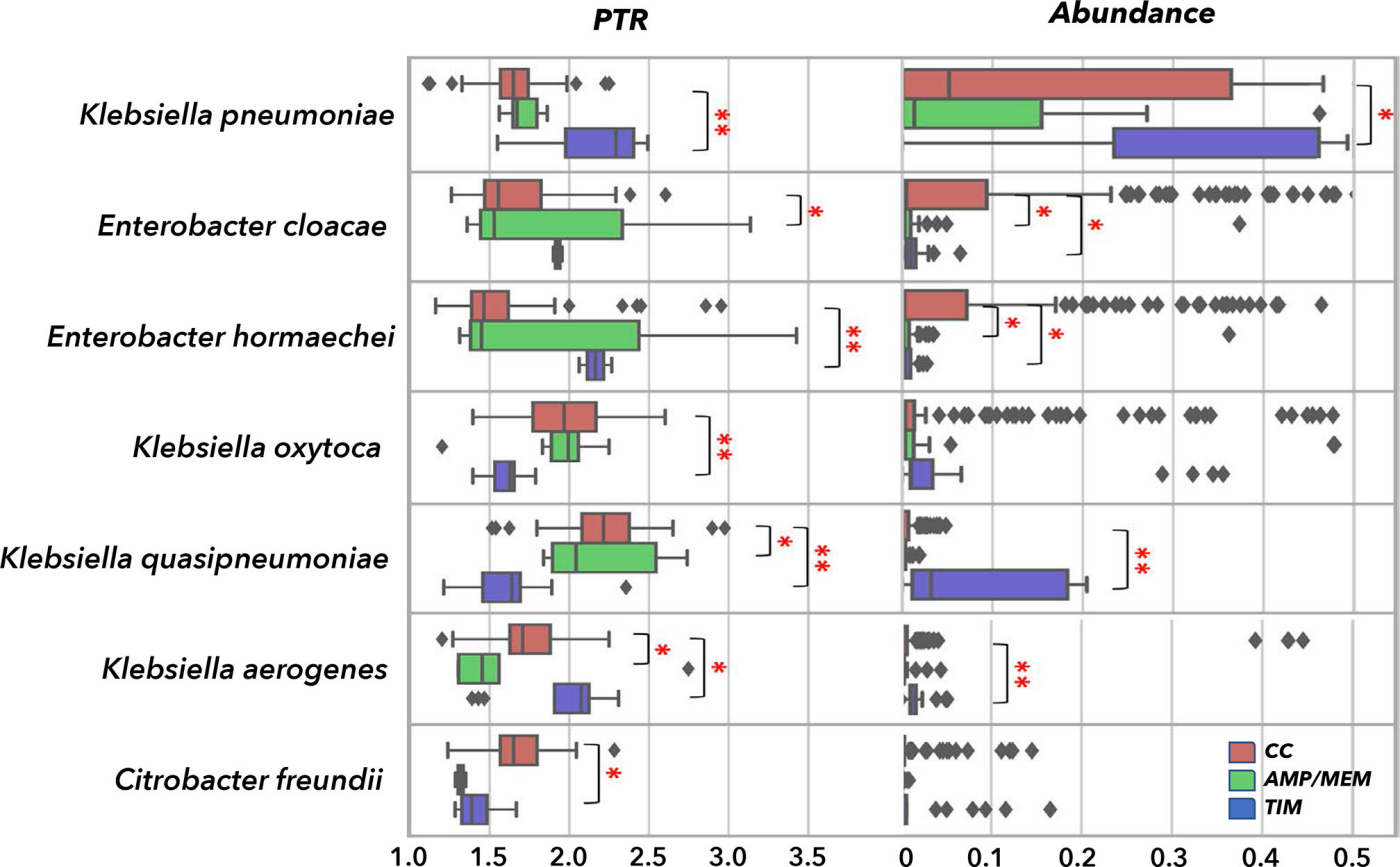
PTR and microbial relative abundance. Box-and-whisker plots of values of bacterial taxa that significantly changed their replication rates after exposure to TIM or AMP/MEM antibiotics. Significant pairs identified with the Mann–Whitney U test are marked with asterisks (*P≤0.05, **P≤0.01).

The bacterial species whose PTR values were affected upon exposure to TIM, MEM or AMP were further investigated (Fig. 5). Since AMP and MEM are both *β*-lactams and elicit similar PTR responses, these were combined into a single group. The taxa with increased replication in the presence of TIM were *K. pneumoniae, Enterobacter hormaechei* and *K. aerogenes *. Actively growing *K. pneumoniae* were also overabundant in the AC. *K. aerogenes* had low relative abundance in the CC, but significantly increased relative abundance and replication rate after TIM exposure (AC). The PTR values dropped for *K. oxytoca, Citrobacter freundii and K. quasipneumoniae* after TIM exposure, while their relative abundance increased. In addition, AMP exposure lowered the relative abundance and decreased the replication rate for *K. aerogenes, Enterobacter cloacae and K. quasipneumoniae*. The relative abundance of *Enterobacter hormaechei* decreased significantly to almost nil after TIM and AMP/MEM antibiotics.

### Resistance genes associated with TIM

PLS-DA and Mann–Whitney U tests were used to identify specific ABR genes that contribute the most to differentiating the resistomes of the TIM from the AMP/MEM group. The PLS-DA was trained to distinguish two groups: infants recently administered AMP/MEM and the TIM group. Ten-fold cross-validation was employed to avoid overfitting the PLS-DA model [70], resulting in a low error rate of 0.16.

**Fig. 6.**
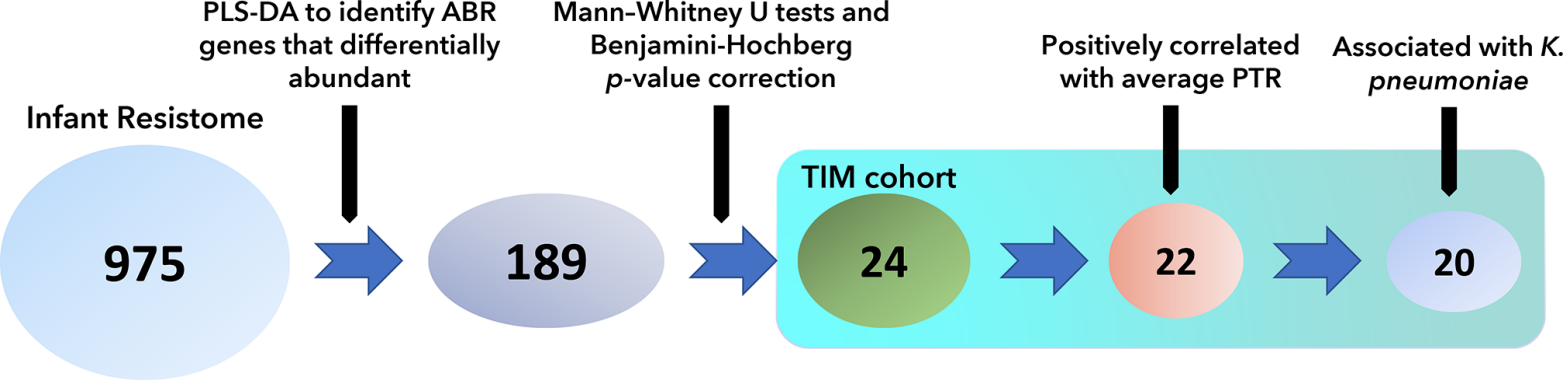
ABR genes associated with TIM exposure. The ABR genes present in the samples from infants treated with TIM. Each circle includes the total number of ABR genes identified at each analysis step, while the text above the blue arrows summarizes the analysis performed at those steps: 1) PLS-DA to identify genes that are differentially abundant between AMP/MEM vs. TIM cohorts; 2) Mann-Whitney U tests and Benjamini-Hochberg *P*-value correction for each gene; 3) Selecting genes positively correlated with TIM; 4) Selecting genes associated with *K. pneumoniae.*

The PLS-DA identified 189 ABR genes that differentiated the AC and CC (Fig. 6). Using the Mann–Whitney U test and Benjamini–Hochberg *P*-value correction for each gene, the number was reduced from 189 to 24 (Supplemental file 1, available in the online version of this article). Of these, only 22 ABR genes were positively correlated with the average PTR values of the TIM group. Twenty of the ABR genes were associated with *K. pneumoniae* (Table 1). These genes were either isolated initially from *K. pneumoniae* [71] or present in at least 30 % of all whole-genome shotgun assemblies available from NCBI for this species, as annotated in CARD [47]. These 20 genes may trigger the rapid replication of the dominant pathogen, *K. pneumoniae *, during TIM exposure (Fig. 6). Most of the ABR genes associated with the TIM response correlated with PTR values, including variants of *K. pneumoniae* OKP chromosomal *β*-lactamase (12 out of 22), followed by MFS efflux pump (*kpnF*, *kpnG* and *kpnE*), RND efflux pump (*oqxA*, *oqxB* and *acrA-acrB-tolC*) and other gene families (*arnA, fosA5* and *ompK37*).

**Table 1.** ABR genes that positively correlate with replication rates and TIM treatment

ABR gene family	Genes correlated with average PTR*
* K. pneumoniae* OKP chromosomal *β*-lactamase	***bla***_**OKP.B.1**_ **,** ***bla***_**OKP.B.2**_ **,** ***bla***_**OKP.B.3**_ **,** ***bla***_**OKP.B.4**_ **,** ***bla***_**OKP.B.5**_ **,** ***bla***_**OKP.B.6**_ **,** ***bla***_**OKP.B.7**_ **,** ***bla***_**OKP.B.8**_ **,** ***bla***_**OKP.B.9**_ **,** ***bla***_**OKP.B.17**_ **,** ***bla***_**OKP.B.20**_ **,** ***bla***_**SHV.79**_
Major facilitator superfamily (MFS) antibiotic efflux pump	***kpnF*** *, ***K. pneumoniae kpnG*** **,** ***K. pneumoniae kpnE***
Resistance-nodulation-cell division (RND) antibiotic efflux pump	**o*qxA*** *, oqxB, K. pneumoniae acrA*-actB-tolC
Other	arnA*, fosA5*, **K. pneumoniae ompK37**

*Genes marked with asterisks are predicted to be growth-enhancing based on causality analysis. Genes associated with *K. pneumoniae* are shown in bold type.

### Identifying potential causal relationships

**Fig. 7.**
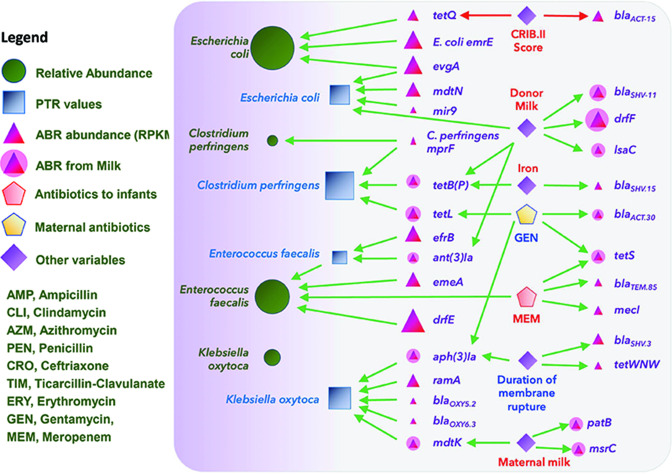

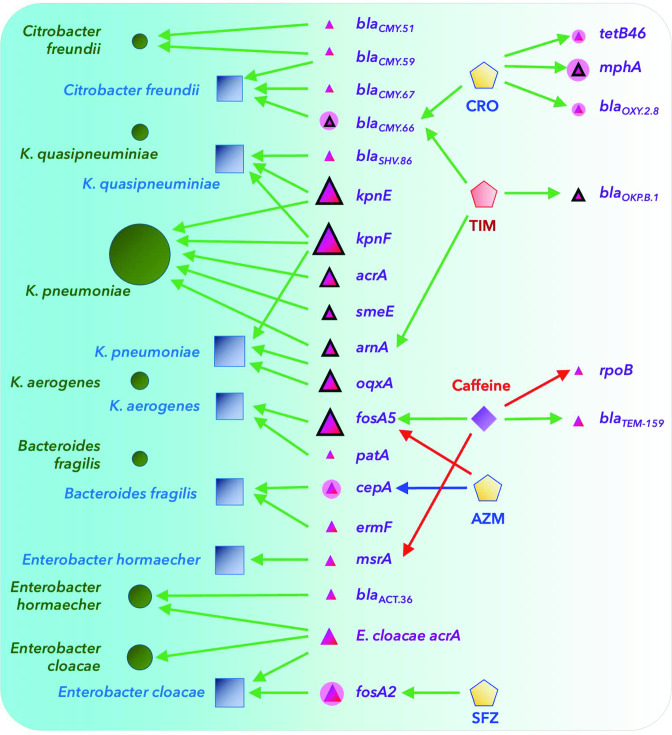

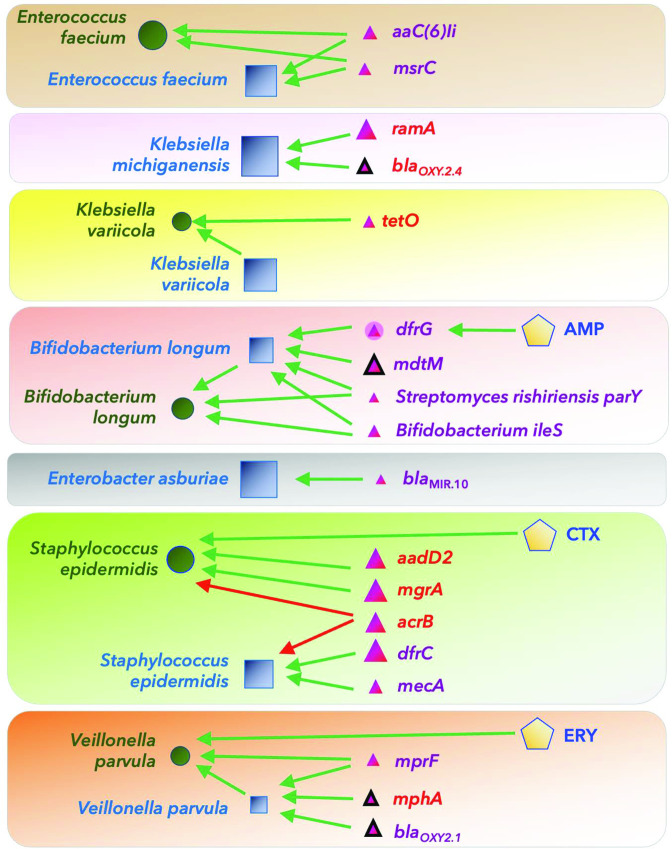
Causal network for the infant gut microbiome. Node sizes are proportional to the average value. Nodes correspond to eubacterial relative abundance (circles), PTR values (rectangles), the abundance of ABR genes (triangles), antibiotic dosage administered to infants (pink pentagons) or mothers (yellow pentagons), and various environmental factors (diamonds).

The predictive causal discovery tool using Bayesian Networks was applied to identify complex relationships among the different entities of the microbiomes in the infant gut samples (Fig. 7). The analysis was performed with the relative abundance and replication rates (PTR) of the 25 most abundant taxa (Fig. 2), combined with the 479 most abundant ABR genes from the resistome profiles, along with 29 infant and 12 maternal variables (see section “Cohort analysis and clinical variables”). A directed arrow between two vertices indicates an inferred direct causal relationship (green and red for positive and negative relationships, respectively). Isolated nodes without any outgoing and incoming causal links were removed. As a result, seven of the 25 taxa were automatically excluded leaving 18 taxa of importance (Fig. 7).

The most influential resistance genes encoded *β*-lactamases (ACT.36, SHV86, SHV147, CMY.51, CMY.59, CMY.66, CMY.67, MIR.9, MIR.10, ACT.5, ACT.36, OKP.B.5, OXY.2.4 OXY.5.2, OXY.6.3, OXY.2.10 and *cepA*), efflux pumps (RND family – *acrA*, *acrB*, *ramA, smeE* and *oqxA*; MFS family – *kpnF, kpnE, mdtM, mdtN, evgA* and *tetL*; ABC family – *efrB, patA, mgrA, tetB, msrA* and *msrC;* MATE family – *emeA,* and *mdtK*; SMR efflux pump – *emrE*)*,* and resistance genes [trimethoprim – *dfrC*, *dfrE* and *dfrG*; defensin – *mprF*; aminoglycoside – *aph(3')-Ia* and *ant(6')-Ia*; aminocoumarin – *parY*; fosfomycin – *fosA2* and *fosA5*; macrolide – *mph*; methicillin – *mecA*; tetracycline – *tetQ*, *tetB(P), tetO*; mupirocin – *ileS*; polymyxin – *arnA*] (Table 2). The only infant variables of significance were dietary information [iron, caffeine and milk (maternal, donor or formula)], antibiotic use (MEM and TIM) and their CRIB II score (Fig. 7). The maternal variables of significance were the duration of membrane rupture and the use of antibiotics, namely AMP, CLI, CTX, SFZ, AZM, CRO, ERY and GEN (Fig. 7).

**Table 2.** Predicted provenance of ABR genes from the causal network with confirmations from existing databases

Genus (phylum)	Species	Predicted ABR genes*
* Bacteroides* (B)	* Bacteroides fragilis *	ermF*(123), cepA*(124)
* Bifidobacterium* (A)	* Bifidobacterium longum *	Bifidobacterium ileS*(125), *mdtM*, *dfrG, Streptomyces rishiriensis parY*
* Citrobacter* (P)	* Citrobacter freundii *	*bla*_CMY.51_ *(126), *bla*_CMY.66_ *(126), *bla*_CMY.67_ *(126), *bla*_CMY.59_
* Clostridium* (F)	* Clostridium perfringens *	Clostridium perfringens mprF*(97.09), tetL, tetB.P*(30.77)
* Enterobacter* (P)	*Enterobacter absuriae*	*bla*_MIR.10_
	* Enterobacter cloacae *	Enterobacter cloacae acrA*(127), fosA2*(128)
	* Enterobacter hormaechei *	*bla*_ACT.36_*(129), *Enterobacter cloacae acrA* [1.52], *msrA*
	* Enterobacter roggenkampii *	*bla*_MIR.10_
* Enterococcus* (F)	* Enterococcus faecalis *	dfrE*(95.74), efrB*(95.87), emeA*[99.25], *ANT(3'')-IIa*
	* Enterococcus faecium *	aac(6)Ii*(90.6), msrC*(60.42)
* Escherichia* (P)	* Escherichia coli *	Escherichia coli emrE*(57.42), *tetQ*, evgA*[35.15], *bla*_MIR.9_, mdtN*[35.2]
* Klebsiella* (P)	* K. aerogenes *	arnA, fosA5*[86.9]
	* K. michiganensis *	*ramA, bla_OXY.2.4_*
	* K. oxytoca *	*ramA_,_ bla_OXY.5.2_ (130)_,_ bla_OXY.6.3_ (130), mdtK,* ANT(3'')-IIa[6.25]
	* K. pneumoniae *	arnA, smeE, acrA*(131), kpnE*(111), kpnF*(111), fosA5(14.57), oqxA*[92.58]
	* K. variicola *	*tetO*
* Staphylococcus* (F)	* S. epidermidis *	acrB, mgrA[15.56], ANT(4'')-I*[24.08], mecA*[54.58], dfrC*(132)
* Veillonella* (F)	* V. parvula *	*mphA, bla_OXY.2.10_, mprF*


*Genes marked with asterisks were validated by:

(%)NCBI WGS prevalence (percentage of NCBI assemblies of predicted species to have at least one hit to a given gene).

(#)literature claiming strong association of the given gene with predicted species. Abbreviations used in the first column: (A): *Actinobacteria*; (B): *Bacteroidetes*; (F): *Firmicutes*; and (P): *Proteobacteria*.

The Bayesian network shows only a few entities directly affected by antibiotic dosage administered to infants. The network suggests that MEM may have causally resulted in the increase of *Enterococcus faecalis *, *tetS*, *mecI* and *bla* _TEM.85_ . Also, the relative abundance of *bla* _CMY.66_ *, arnA* and *bla* _OKP.B.1_ genes may have significantly increased due to administration of TIM to infants and CRO to the mother.

The causal network suggests that only a few ABR genes were affected by antibiotic dosage. The Mann–Whitney U test identified significantly abundant genes after specific antibiotic treatments. The relative abundance of resistance genes *arcA, kpnF, oqxA, arnA, fosA5, bla* _OXY_ _.2.4_, *bla* _OXY_ _.2.10_, *bla* _OKP.B_ _.5_ *, bla* _CMY.66_ , *mdtM, mphA* and *smeE* was significantly higher in infants with TIM administered within the last 48 h before sampling (Fig. 7). Many of these resistance genes (*fosA5, oqxA, kpnF, kpnE, smeE, arnA* and *acrA*) had a direct edge to *K. pneumoniae* PTR or relative abundance (Table 3).

**Table 3.** *
K. pneumoniae* resistome in the infant gut microbiome predicted by Bayesian network analysis

Family	Components	Confer resistance
PMR phosphoethanolamine transferase	*arnA*	Peptide
RND	*smeE, acrA, oqxA*	Macrolide, fluoroquinolone, tetracycline, phenicol, cephalosporin, glycylcycline, penam, rifamycin, diaminopyrimidine, nitrofuran
MFS	*kpnE, kpnF*	Macrolide, aminoglycoside, cephalosporin, tetracycline, peptide, rifamycin
Fosfomycin thiol transferase	*fosA5*	Fosfomycin

According to the causal Bayesian Network (Fig. 7), the genes encoding β-lactamase, *bla*_CMY-51_, *bla*_CMY-59_, *bla*_CMY-66_, and *bla*_CMY-67_ may have contributed to the survival of *Citrobacter freundii *. A path TIM →*bla*_CMY-66_→ PTR of *Citrobacter freundii* and TIM →*arnA→* relative abundance of *K. pneumoniae* suggests that *bla*_CMY-66_ and *arnA* were particularly effective against TIM and probably play a role in the proliferation of the two opportunistic pathogens, *C. freundii* and *K. pneumoniae *.

Maternal milk has a positive link to the relative abundance of the *mdtK* encoding efflux pump that confers resistance to norfloxacin, doxorubicin and acriflavine whereas the donor milk associates with an increase in many resistance genes (*tetB*, *lsaC, ant(3)la, drfF* and *bla*_SHV-11_) in the infant’s gut (Fig. 7). The use of maternal AMP, SFZ, CLI, PEN, CRO and GEN appears to contribute to the increased relative abundance of various resistance genes [AMP to *dfrG*; SFZ to *fosA2*; AZM to *cepA*; CLI to *efmA*; PEN to *tetB*; CRO to *bla*_CMY66_, *bla*_OXY.2.8_, *bla*_OKP.B.1_ *tetB*(46), and *mphA*; and GEN to *aph(3)la, tetS, tetL* and *bla*_ACT.30_].

In cases where there is a causal link inferred from an ABR gene to both PTR and relative abundance of the same taxon, it suggests higher confidence in the role of that resistance gene in bacterial growth. Such relationships included *ileS and parY to Bifidobacterium longum*, *msrC* and *aac(6)li to Enterococcus faecium, bla*_CMY-59_ to *Citrobacter freundii, evgA* to *Escherichia coli *, *mprF* to *Clostridium perfringens *, *arnA* to *K. pneumoniae *, *acrA* to *Enterobacter cloacae* and *mprF* to *Veilonella parvula*.

## Discussion

This work presents the MeRRCI pipeline that extends the standard metagenomic analysis by integrating the metagenome, metaresistome and metareplicome into a unified causal inference framework. This work is the first to use causality to infer antimicrobial resistance mechanisms from metagenomic studies. As proof-of-concept, MeRRCI was applied to infant gut microbiome data to determine microbial survival strategies against antibiotics.

### The scalable MeRRCI novel pipeline enhances microbiome analyses

The algorithm is designed for high-performance computing environments providing scalability and computational efficiency, as previously shown by its progenitor, FLINT [42]. MeRRCI can handle a larger number of samples and a much greater number (than previously possible) of associated sequencing reads using a cluster or cloud. In addition, it can be scaled to deal with much larger reference genome collections. Splitting the indexes for the RefSeq reference genomes database into smaller, manageable pieces provides an efficient framework for the scalable computation of eubacterial composition and PTR values. In addition, the associated computation times are shorter, and the costs are meagre if they are performed on a cloud service such as AWS [72]. One can argue that the outcome of MeRRCI is biased towards *Klebsiella pneumoniae* and *Escherichia coli* due to overrepresentation of those strains in RefSeq [73]. To ensure that our findings are not compromised, we compared the relative abundance of those species computed by MeRRCI and the popular metagenomic profiler, Metaphlan 3 [74]. The effect of the database bias should be lower in Metaphlan 3, as species are counted using clade-specific marker genes with minimal overlap, and the raw counts should be higher with a biased database. The two approaches, MeRRCI and Metaphlan 3, had a significant Pearson’s correlation for the top three most abundant species: *Klebsiella pneumoniae* (*r*=0.77, *P*=1e-77), *Escherichia coli* (*r*=0.83, *P*=1e-101) and *Staphylococcus epidermidis* (*r*=0.85, *P*=1e-111). Furthermore, the raw counts from Metaphlan 3 were, more often than not, higher than that arrived at by MeRRCI. Therefore, we conclude that bias towards species with over-represented sequences in RefSeq did not significantly affect our dataset. The MeRRCI code repository includes the source code of this experiment.

The standard analyses of microbiome compositional profiles were enhanced using the information on instantaneous dynamics evidenced by inferred replication rates. The MeRRCI estimate of replication rates for each taxon (replicome) provides valuable information on eubacterial dynamics inferred from the static snapshots of metagenomic samples. The MeRRCI pipeline uses extensive genome collections, which allowed us to measure PTR values for many more strains present in the samples. In contrast, existing tools for computing PTR values, such as GRiD, use curated reference genome collections with one genome per species [26]. GRiD is likely to lower the computation sensitivity of the PTR profiles since strains not represented in the reference collection may have very different replication rates. A recently developed SMEG tool deals with strain variations by finding a representative reference sequence [27]. However, it was impossible to parallelize SMEG in any obvious way, thus making it computationally slower than the tools experimented with. Other tools for computing PTR values, such as iRep [20] and Demic [75], use draft-quality metagenome-assembled genomes. Consequently, the computed PTR values are approximate averages across multiple strains since reconstructed genomes often have to be dropped because of insufficient coverage. An alternative method using codon usage bias (CUB) can estimate maximal growth rates (i.e. growth potential) based on highly expressed bacterial genes relative to the rest of the genome [76, 77]. While CUB can accurately distinguish fast and slow-growing organisms, the measures lack per-sample granularity [78]. Even though this approach has been extended to metagenomes, it estimates relative ‘growth potentials’ based on genomic content and is not a reflection of true growth rate values under specific conditions. Consequently, it cannot sense the dynamics of growth rates of the same bacterial taxon under different conditions (e.g. the presence of antibiotics). On the other hand, PTR values reflect the dynamics and can be computed for every metagenomic sample for each eubacterium and, therefore, are more suitable for computational cause–effect analyses.

A causal discovery algorithm using the Bayesian network [79] was implemented in the MeRRCI pipeline. Causality has shown promising results in inferring complex relationships among different entities in a microbiome [80, 81] and differentiating between pathogenic and beneficial bacteria using taxon relative abundance information [36]. Furthermore, inference of causal structures from observational studies using *directional separation* (or just *d-separation*) is based on conditional independence tests [57]. In addition, imposing a set of constraints based on a biological understanding of the microbiome and its interactions with the host informs the PC Stable algorithm to incorporate only allowable edges. These criteria allow differentiation between causal relationships and those that are merely correlational or occurring by chance. To the best of our knowledge, this work is the first to use causality to infer significant ABR genes in the metagenome, the associated microbes, and clinical variables that influence a microbiome and health outcomes.

### Eubacterial relative abundance is not correlated with replication rate

The MeRRCI pipeline analyses show that a higher replication rate does not imply higher relative abundance as previously reported [16, 20, 22] (Fig. 2). This claim is further supported by the casual analyses, where only a few edges are going from bacterial replication (squares) to taxon relative abundance (circles) (Fig. 7), suggesting a weak causal link between them. In eight out of 25 taxa, relative abundance correlates negatively with PTR; in three species (*Bifidobacterium longum, Enterococcus faecium* and *K. michiganensis *), the correlation was positive in both the AC and CC (Fig. 2a). However, the correlation was insignificant in the remaining 19 species (Fig. 2a). The weak correlation may be because measured bacterial relative abundance is a net result of replication and death. DNA replication is necessary for bacterial growth but not sufficient since dormancy and death can impact growth. The computed replication rate represents the proportion of bacteria participating in cell division [16, 19]. In addition, a recent study showed that absolute bacterial growth in the ocean microbiome only weakly correlates with PTR values [78]. Thus, bacterial relative abundance, which is computed as part of standard metagenomic profiling, cannot be used as a *proxy* for replication rates or vice versa. More experimental studies are needed for an exact interpretation of PTR values and should be cautiously applied in computational microbiome investigations.

### Administration of β-lactams may adversely affect the gut microbiome

This study showed that the administration of β-lactams to preterm infants lowered the replication rate in some taxa and increased it in others. The increase was seen only in eubacterial species that are known to harbour ABR genes. The lowest replication rate was observed with MEM, a carbapenem (Fig. 4). This family of antibiotics targets dividing cells disrupting replication [82]. Thus, this finding further validates the MeRRCI pipeline.

The causal network shows a direct (green) link from MEM to the relative abundance of *Enterococcus faecalis *, suggesting that MEM is ineffective against this pathogen, leading, in fact, to proliferation. MEM has been previously reported to be ineffective against this pathogen [83]. *Enterococcus faecalis* is a known early gut colonizer [84]. Enterococci are also a leading cause of hospital-acquired infections [85]. However, an antibiotic treatment that contributes to an increase in the relative abundance of enterococci will lead to a reduction in the protective commensals, and an increase in *Clostridium* species [86].

The use of antibiotics may permanently alter the original microbiota, causing cumulative dysbiosis upon subsequent exposure [87–90]. β-Lactams cause the highest dysbiosis of the gut microbiome [91]. The surviving bacteria will probably proliferate upon MEM removal and contribute to the emergence of resistance, as previously demonstrated [92]. Though carbapenems are last-resort antibiotics with guidelines for prudent use in humans and animals, carbapenem-resistant infections are on the rise [93]. Furthermore, carbapenems can exacerbate the already dire health contributing to *Clostridioides difficile *-associated diarrhoea (CDAD), a condition too often seen among preterm infants [94, 95].

### A high relative abundance of pathobionts in premature infants is an unintended consequence of antibiotic treatment

The presence of ABR genes can contribute to the PTR and relative abundance of specific taxa that can adversely impact the health of preterm infants. A direct edge from an ABR gene to taxon relative abundance or PTR was surmised to suggest that the taxon was the expected source of that particular resistance gene (Table 2 and Fig. 7). Due to horizontal gene transfer, it will not always be possible to precisely infer the microbe that harbours specific ABR genes. Our pipeline predicted the most likely bacterial source of 56 ABR genes (Table 2), from which 18 were previously observed in NCBI WGS sequences and 15 were isolated in the lab by other researchers [96–106]. The CARD database has annotations to studies that indicate the primary source of many ABR genes [44]. The CARD database also provides prevalence values giving the percentage of whole-genome shotgun assemblies of the taxon from the NCBI repository with at least one hit to the predicted ABR gene [44].

Most dysbiosis events result in reducing beneficial bacteria such as *Bifidobacterium longum *. Our study did not show a significant change in *Bifidobacterium longum* relative abundance after antibiotic administration (Fig. 2a), and the computational causal graph highlighted four ABR genes important for the species' survival: *dfrG*, *mdtM*, *parY* and *ileS* (Fig. 7). *Bifidobacterium longum* is used as a probiotic to alleviate gastrointestinal, immunological and infectious diseases and has been recommended for use in ulcerative colitis and coeliac diseases [107].

A causal link from an ABR gene to both PTR and relative abundance suggests higher confidence in the role of that resistance gene in bacterial growth. The replication rate of other pathogens such as *K. aerogenes* and *Enterobacter hormaechei* also increased after TIM administration (Fig. 5). It appears that the potential proliferation of opportunistic pathogens such as *Klebsiella* species, in particular *K. pneumoniae* (discussed below)*, Citrobacter freundii, Clostridium perfringens, Enterococcus faecium* and *Enterococcus faecalis* is in part driven by the presence of the ABR genes in the microbiome, presumably in their genomes. The causal graph also highlighted several opportunistic pathogens, such as *Veilonella parvula* (*mprF, mphA* and *bla_OXY.2.1_*)*, Staphylococcus epidermidis* (*mecA*, *dfrC, mgrA* and *aadD2*) and *Citrobacter freundii* (*bla_CMY_* alleles); these three species are well-known aetiological agents of hospital-acquired infections that can lead to pulmonary infections, meningitis and sepsis in neonates [108–110]. It is also important to note that the use of TIM and CRO in preterm infants and mothers, respectively, influences the relative abundance of *Citrobacter freundii,* further arguing against the use of these antibiotics.

The causal graph does not show any negative impact of antibiotics on relative abundance values. Instead, it may be an artefact resulting from including only major bacterial taxa present in at least 5 % of the samples with reading coverage of 5×. This inclusion criterion is likely to have biased the analysis to include more antibiotic-resistant species, thus explaining the lack of direct impact of antibiotics. It is also important to note that hidden confounders reduce model sensitivity, such as data sparsity, high dimensionality, errors in metagenomic sequencing and read mapping.

### Despite low relative abundance, *Clostridium perfringens* has the highest replication rate

Despite its low relative abundance*, Clostridium perfringens* was the most highly replicating species in both cohorts (Fig. 2a-b). From the causal graph, it appears that the presence of the *mprF* gene contributed to the survival of *Clostridium perfringens *. The *mprF* gene encodes resistance to host cationic antimicrobial peptides (CAMPs) and antibiotics such as vancomycin, methicillin and gentamycin [111]. MprF has been recommended as a therapeutic target, as inactivation of this protein leads to additional susceptibility to daptomycin, vancomycin, methicillin and gentamycin. Additionally, a positive edge from *tetL* and *tetB(P*) to PTR of *Clostridium perfringens* suggests that those genes might confer resistance to tetracycline. Based on a previous study, strains of *Citrobacter freundii* are of significant concern in the paediatric population and can cause serious neonatal infections [112].

By its exponential replication, *Clostridium perfringens* has the potential to dominate and be harmful. *Clostridium perfringens* has been implicated in various human illnesses, including gas gangrene, food poisoning and necrotizing colitis [113, 114]. It is well known that necrotizing colitis is a significant cause of morbidity and mortality in the preterm infant population [115]. However, other factors, such as intrapartum complications, respiratory distress syndrome, congenital disorders, sepsis, pneumonia and meningitis are additional causes of neonatal death [116, 117].

### TIM exposure may contribute to the *K. pneumoniae* outgrowth

The causality analysis highlighted the most influential ABR genes and clinical variables for multi-drug-resistant ESKAPE pathogens, including *K. pneumoniae* [118]. The combination of beta-lactamase inhibitors such as clavulanate with β-lactams (as in the case of TIM) significantly impacts the relative abundance and replication rate of *K. pneumoniae* (Fig. 5). The average PTR is close to 2 after it was administered, indicating that the dominant taxon was replicating even after the drug exposure. Multiple ABR genes conferring multi-drug resistance to *K. pneumoniae* were correlated with the replication rates (Table 1). *K. pneumoniae* is intrinsically resistant to many *β*-lactams due to the expression of a chromosomally encoded *β*-lactamase constitutively conferring resistance to ampicillin, amoxicillin, carbenicillin and ticarcillin, but not to extended-spectrum *β*-lactams. Three groups of *bla* genes – *bla* _SHV_ , *bla* _OKP_ and *bla* _LEN_ – evolved in parallel. *K. pneumoniae* strains harbouring *bla* _OKP_ are common in acute care settings and serve as a reservoir for this gene [119, 120].

The resistance genes, *fosA5, oqxA, kpnE, kpnF, acrA, arnA* and *smeE*, had a causal effect on the relative abundance and correlated with PTR and/or replication rate of *K. pneumoniae* (Table 3). *K. pneumoniae* harbours many genes that confer multidrug resistance, which include those encoding efflux pumps (*oqxAB, kpnEF*) [96, 121], outer membrane porin protein (*ompK37*) [122], *acrA-tolC* (confers colistin resistance) [123],and *fosA* (a plasmid-encoded gene that confers fosfomycin resistance) [124]. The *K. pneumoniae kpnEF* system confers resistance to cefepime, ceftriaxone, colistin, erythromycin, rifampin, tetracycline and streptomycin [96]. The expression of *oqxAB* genes is also common in clinical isolates of *Enterobacteriaceae* and *K. pneumoniae *, conferring low to intermediate resistance to quinoxalines, quinolones, tigecycline and nitrofurantoin [121]. Loss of *ompK37* in *K. pneumoniae* confers resistance to cefoxitin and expanded-spectrum cephalosporins [122, 125, 126]. The *fosA* encoding fosfomycin-modifying enzyme of plasmid origin was identified initially in *Serratia marcesens* [127, 128]. Another gene predicted to positively affect the relative abundance of *K. pneumoniae* was *smeE* (Fig. 7), which is the RND protein of the efflux complex *smeDEF* found in *Stenotrophomonas maltophilia* [129]. Even though none of the *K. pneumoniae* shotgun assemblies available in NCBI has *smeE* in their genome [44], the gene could have been acquired through horizontal gene transfer.

The causal network predicted that *K. pneumoniae* has the necessary resistome not just to survive but to thrive after TIM treatment. *K. pneumoniae* is a well-known pathogen associated with an increased mortality rate in preterm infants [130]. It is indeed a considerable concern, leading us to conclude that the use of TIM for premature infants should be discouraged, perhaps after further laboratory verifications.

### Milk is a potential source of ABR genes

The causal analysis strongly suggests that the mother or donor milk affects the taxon relative abundance and resistome present in the infant’s gut (Fig. 7), supporting the previous claim that resistance genes are passed on to infants through human milk [131]. The presence of ABR genes in cow's milk has been demonstrated [132]. Though we lack the mother’s microbiome data, it is not far-fetched to suggest that human milk may be the source of the resistance genes in the infant gut microbiome.

## Conclusion

Overall, the MeRRCI pipeline presented here provides a comprehensive view of the eubacterial component of the microbiomes and infers the causal relationships between variables from the metagenomic, metareplicomic and metaresistomic profiles. Our data suggest that species may increase or decrease their replication upon antibiotic administration dictated by the antibiotic type and resistance genes present within the microbiome. The causal analysis highlights the most critical resistance genes and environmental factors that help bacteria survive antibiotic exposure. This study demonstrates that a high relative abundance of pathobionts that lead to many acute and adverse long-term sequelae in premature infants is an unintended consequence of the current antibiotic regimen. In particular, the use of TIM or β-lactams should be revisited, and perhaps aminocoumarin should be considered as an alternative that would allow good bacteria such as *Bifidobacterium longum* to compete in the environment.

The results of causal analysis led to compelling hypotheses that need to be validated in the laboratory. Errors in computational inference methods may be caused by several factors, including small sample size, errors in sequencing, errors in mapping reads to the correct genomes, errors in the inferred relative abundance matrix, presence of undocumented or new bacterial taxa, and, most importantly, the inability to measure known or hidden confounders related to the diet and environment of the mother and infant, and much more. Nevertheless, we have set the stage for a novel approach to studying microbiome dynamics from studies with limited time points.

## Supplementary Data

Supplementary material 1
